# Cross-Cultural Translation of the nChinese Version of Pain Care Quality Surveys (C-PainCQ)

**DOI:** 10.31372/20190404.1072

**Published:** 2020

**Authors:** Jia-Wen Guo, Hui-Ying Chiang, Susan L. Beck

**Affiliations:** a University of Utah, United States; b Chi Mei Medical Center, Taiwan

**Keywords:** cultural, pain, professional–patient relations, quality of health care, surveys and questionnaires, translations

## Abstract

Health disparities in pain care continue to exist among non-English-speaking Chinese-Americans. The Pain Care Quality?© (PainCQ) surveys, a valid instrument measuring the quality of pain care from the patient’s perspective, is available only in English currently. This study generated a Chinese version of the PainCQ (C-PainCQ) following a cross-cultural translation approach to address health equity in pain care. A multicultural, bilingual expert team produced a good quality, prefinal version of C-PainCQ. Chinese-speaking patients (*n* = 55) evaluated conceptual and content equivalence while bilingual participants (*n* = 13) reviewed semantic equivalence of C-PainCQ items. Feedback from participants, including adding a new item related to education on medication compliance, was used to revise the tool. This C-PainCQ is ready for future research to examine the reliability and construct validity with a large sample of Chinese-speaking patients.

## Introduction

“Eliminate health disparities, achieve health equity, and attain health literacy to improve the health and well-being of all” is one of the overarching goals proposed in Healthy People 2030 (U.S. Department of Health and Human Services, 2018). Health disparities in pain care continue to exist among ethnically diverse groups, including non-English-speaking Chinese-Americans or patients from Chinese background (Xu, Luckett, Wang, Lovell, & Phillips, 2018). Culture influences pain experiences and management in Chinese patients (Tung & Li, 2015; Xu et al., 2018). Having a better understanding of the quality aspects of pain care among this population is an important initial step before developing appropriate cultural approaches to improve their pain care.

The Pain Care Quality © (PainCQ) surveys, consisting of two surveys, *Interdisciplinary Care* Survey (PainCQ-I) and *Nursing Care Survey* (PainCQ-N), using a Likert-type scale (from 1 – *strongly disagree* to 6 – *strongly agree*), were developed and tested for rating the quality of pain care from the patient’s perspective (Beck, Towsley, Berry, Brant, & Smith, 2010; Beck, Towsley, Pett et al., 2010; Pett et al., 2013). PainCQ-I contains 11 items and two subscales: *Partnership with the Health Care Team* measuring care related to collaboration among health care team members and with the patient, and *Comprehensive Interdisciplinary Pain Care* measuring care focusing on the whole person and how pain influences patients’ relationship and activities. The PainCQ-N contains 22 items with three subscales: *Being Treated Right* representing care provided by concerned nurses who understand and respond promptly patient’s pain, *Comprehensive Nursing Pain Care* reflecting pain management provided by nurses including patient education and non-pharmacological approaches, and *Efficacy of Pain Management* indicating patient comfort results from medications that work effectively and quickly. Exploratory and confirmatory factor analysis support the validity of the surveys (Beck, Towsley, Pett et al., 2010). Evidence supports the reliability of the PainCQ surveys; Cronbach’s alphas range from .76 to .95 for all subscales (Beck, Towsley, Pett et al., 2010) and has been used in evaluating the quality of pain care in studies (Beck et al., 2016; Beck et al., 2019; Rice et al., 2019); however, currently this instrument is available only in English.

## Background

### Cross-Cultural Translation Methods

To translate a measure from one language or culture to another, a cross-cultural approach is necessary to ensure that key latent constructs are presented appropriately across languages or cultures. Beaton, Bombardier, Guillemin, and Ferraz (2000) proposed a cross-cultural translation approach with six steps: (1) initial translation (from the source to target language), (2) synthesis of the translations (from Step 1), (3) backward translation (from the target to source language), (4) expert committee review (producing a prefinal version), (5) testing of the prefinal version, and (6) approval of the final translated version. This method has been widely used to generate culturally compatible translations of self-report measures (Albach, Wagland, & Hunt, 2018; Wang et al., 2017).

### Common Issues of Translating From English to Chinese

Chinese and English are different both at the linguistic (e.g., characters, grammatical structures) and at the cultural level. Challenges have been identified in translating items from English into Chinese; for example, it is common to use passive voice in English but not in Chinese; Chinese verbs have no tense but English verbs do (Guo, 2016; Tsai, Luck, Jefferies, & Wilkes, 2018). In Chinese, additional words (e.g., “現在” means *now*) are required to indicate the proper tense. Moreover, Chinese characters can be written or presented as traditional (e.g., for Taiwan or Hong Kong) or simplified Chinese (e.g., for China and Singapore). For example, body in traditional Chinese is “體” having 23 strokes and in simplified Chinese is “体” having 7 strokes; both Chinese characters are pronounced the same as /tǐ/. Although the pronunciation of a given Chinese character is the same for both traditional and simplified Chinese, the word may have different meanings due to cultural differences. For example, “土豆” is pronounced as /tǔ dòu/, which pronounces the same in both traditional and simplified Chinese; but it means *peanuts* for people from Taiwan but means *potatoes* for those from China.

To address these differences in Chinese characters and cultures, some translated surveys have more than one Chinese version. For example, the Brief Pain Inventory (BPI) has two Chinese versions: Taiwanese version (BPI-T) (Ger, Ho, Sun, Wang, & Cleeland, 1999) and Chinese version (BPI-C) (Wang, Mendoza, Gao, & Cleeland, 1996). The translated contents of both versions are very similar and the main difference between them is that BPI-T is presented in traditional Chinese while BPI-C is presented in simplified Chinese. Hsu, Lu, Tsou, and Lin (2003) used the BPI-C for Taiwanese participants by just changing simplified Chinese to traditional Chinese; the Cronbach’s α for the BPI-C in that study indicated good internal consistency. It indicated that it may be unnecessary to have many Chinese versions for the same translated survey; one with good cross-cultural adaptation may be practical for use among Chinese speakers. Therefore, our aim was to generate one Chinese version of the PainCQ (C-PainCQ) surveys that was valid cross-culturally among all Chinese speakers.

## Methods

### Study Design

We followed the approach for cross-cultural adaptation of self-report measures (Beaton et al., 2000); the six steps are listed below.

**Step 1: Forward translation.** Two bilingual translators with graduate degrees, one from Taiwan and the other from China, independently translated the PainCQ surveys to Chinese. Each translator received the translated PainCQ surveys (from English to Chinese) generated by Google Translate as a reference. As suggested by Guo (2016), the Google Translate outcome may not be adequate but can be used to facilitate the survey translation process by providing an initial translation draft to translators.

**Step 2: Synthesis of the forward translation.** The two translations from Step 1 were compared. Discrepancies, such as using different Chinese terms to describe the same concept, between the translations were addressed via discussion between the translators until consensus was reached. The discussion was held via conference call and hosted by the author (JG). The first draft of the C-PainCQ surveys was then generated.

**Step 3: Backward translation.** Two bilingual translators with graduate degrees, one from Taiwan (with Chinese as her first language) and one from the United States (with English as his first language), independently completed a backward translation of the first draft of the C-PainCQ surveys to English. Again, the translated C-PainCQ surveys (from Chinese to English) generated by Google Translate was provided to translators as a reference.

**Step 4: Expert committee review.** A multicultural, bilingual expert committee consisted of four translators from the forward and backward translation steps and two nurse researchers (one has expertise in instrument development; the other developed the PainCQ surveys). They met to compare the two backward-translated English versions of the PainCQ surveys with the original PainCQ version to evaluate the accuracy and consistency of the wording and the translation equivalence. The committee resolved any disparities during the meeting and generated the prefinal version of C-PainCQ surveys.

**Step 5: Testing the prefinal version through cognitive interviews.** Three types of equivalence were examined: conceptual (meaning that the measured construct was appropriately translated into Chinese), content (meaning that the translated item was relevant to the Chinese culture), and semantic (meaning that the translated words retained a meaning similar to the meaning in the English version) equivalence. Two groups of Chinese speakers participated. Group 1 participants, (*n* = 55) who were fluent in speaking and reading Chinese and who were currently admitted to the hospital for pain control or who experienced pain during their hospitalization, evaluated conceptual and content equivalences. Group 2 participants (*n* = 13), who were fluent in speaking and reading both English and Chinese, but were not hospitalized nor in pain, evaluated semantic equivalence. The exclusion criterion for both groups was inability to participate in a 30-minute interview. During the interview, Group 1 participants were asked to use their own words to describe the meaning of each item and how the content of each item could be related to their own experiences in pain care; Group 2 participants (all bilingual in English and Chinese) were asked to evaluate whether the translated items were equivalent in meaning to those from the original English version. All the participants were asked to indicate whether any additional items could be added to assess the quality of pain care after they read through all items from the prefinal version of the C-PainCQ surveys. To avoid interview fatigue, each participant was asked to review 11 items; however, the participants could voluntarily review more items. A $25 gift card was offered to each participant for their time. Depending on different regions, people read Chinese in either traditional or simplified version of written Chinese characters. Participants from Taiwan or Hong Kong are reading traditional Chinese and those from China or Singapore are reading simplified Chinese. To accommodate this need, we prepared both traditional and simplified Chinese versions of the prefinal C-PainCQ surveys for our participants. The survey questions were stated and structured exactly the same in both Chinese versions of the prefinal C-PainCQ surveys; the only difference was one in traditional Chinese characters and the other in simplified Chinese characters.

**Step 6: Approval of the final translated version.** The feedback from Step 5 was consolidated to generate a final version of the C-PainCQ surveys for review and approval. The final versions of the C-PainCQ surveys were reviewed and approved by two nurse researchers from the expert committee. In addition, we tested the readability of the final version of C-PainCQ by using Chinese Readability Index Explorer, CRIE 3.0 (http://www.chinesereadability.net/CRIE/), which is a web-based tool for analyzing readability-level for written Chinese materials (Sung, Chang, Lin, Hsieh, & Chang, 2016).

### Ethical Considerations

This study was approved by the University of Utah Institutional Review Board (IRB) (#00075794) and the IRB at Chi Mei Medical Center in Taiwan (#10401-009). Written informed consent was obtained.

## Results

### Translation of PainCQ Surveys

The first four steps were effective in completing the translation process. In Step 2 (synthesis of the forward translation) and Step 4 (expert committee review), we found that the literal translation was adequate and reflected the original meaning of the items in original English version of PainCQ surveys. The use of synonym—whether those similar but different terms expressed the same concept—were common “discrepancies” in the discussion of translation. For example, *healthcare team* was translated “醫療小組” or “醫護圑隊” and both translations were appropriately done; the former was suggested in the prefinal version of C-PainCQ surveys by our expert team based on which one was more commonly used in the context of clinical care.

### Review of the Prefinal Version of the C-PainCQ Surveys

A convenience sample of 68 participants was recruited to evaluate the prefinal version of the C-PainCQ surveys in two groups. To ensure the cross-cultural adoption of the measure, we intentionally included participants from different regions or countries such as Taiwan, China, Hong Kong, or Singapore. Group 1 participants (*n* = 55) were recruited from a medical center in Taiwan (*n* = 40) and the community in Utah, USA (*n* = 15). Group 2 participants (*n* = 13) who were bilingual in English and Chinese were recruited from the community in Utah (Table 1). Each participant was asked to review a minimum of 11 items. Group 1 participants reviewed from 11 to 33 items with a mean of 17; Group 2 participants reviewed all 33 items.

**Table 1:** Demographic Characteristics of the Study Sample (N = 68)

Characteristic	Group 1: *N* = 55 *n* (%)	Group 2: *N* = 13 *n* (%)
Gender		
Female	34 (61.8)	8 (61.5)
Male	21 (38.2)	5 (38.5)
Education level		
Junior high school	17 (30.9)	0
High school	17 (30.9)	0
College	12 (21.8)	5 (38.5)
Master’s degree	3 (5.5)	6 (46.2)
Doctorate	6 (10.9)	2 (15.3)
Employment status		
Unemployed	41 (74.5)	4 (30.8)
Employed	14 (25.5)	9 (69.2)
Religion		
Taoist	28 (50.9)	2 (15.4)
Christian	9 (16.4)	0
Buddhist	5 (9.1)	2 (15.4)
None	13 (23.6)	9 (69.2)
Birth place		
Taiwan	46 (83.6)	5 (38.5)
China	6 (10.9)	6 (46.2)
Hong Kong	2 (3.6)	1 (7.7)
Singapore	0	1 (7.7)
Vietnam^a^	1 (1.8)	0
First language^b^		
Taiwanese	28 (50.9)	0
Chinese	25 (45.5)	11 (84.6)
Cantonese	2 (3.6)	1(7.7)
English	0	1 (7.7)
Residence		
Taiwan	40 (72.7)	0
United States	15 (27.3)	13 (100)

*Note.* Group 1 evaluated conceptual and content equivalences; Group 2 evaluated semantic equivalence. For Group 1, mean age = 52.8 (*SD* = 1.2, range = 36–77). For Group 2, mean age = 39.4 (*SD* = 3.9, range = 23–70).

^a^This participant from Vietnam whose first language is Cantonese spoke Chinese fluently.

^b^Our participants whose first language was not Chinese could speak and read Chinese fluently.

Several findings highlight the evaluation of conceptual, content, and semantic equivalences in the cognitive interviews. First, the participants from both groups expressed that they can comprehend the items from the prefinal version of the C-PainCQ surveys after they read through all 33 items. Second, it was suggested that the translation and concept of *my nurse* be changed to *nurse*. Some participants could not recall that there was a primary nurse during their hospitalization and that they received care from several nurses; therefore, it was suggested that *my* be dropped from the Chinese translation. Third, *healthcare team* was a term that needed some clarification. Our expert team suggested translating *healthcare team* as “醫療小組,” which was understood as expected by most participants, but some participants interpreted it as a team of doctors only, to the exclusion of other healthcare providers. To clarify it, we added the phrase “包括醫生、護士和其他醫療人員,” which means “including doctors, nurses, and other healthcare providers” to explain *healthcare team.* Other changes were made to ensure the same meaning across Chinese speakers. For example, *nurse* can be translated as “護士” or “護理人員”; the former was chosen for the C-PainCQ surveys because the latter is less commonly used and is understood by people from China to mean nurses and unlicensed aides. In another example, for the phrase *positioning my body*,** we chose to use “調整姿勢” instead of “調整體位” because most people from Taiwan may interpret the latter as a sexual position.

Two participants mentioned noticing that their Chinese friends often stopped taking pain medication early or reduced the medication dose because they were afraid of addiction; in consequence, their friends experienced more pain. It was thus suggested to add an item related to education on medication compliance (See Item 1.12, Table 2) to the C-PainCQ Interdisciplinary Care survey.

**Table 2:** English-to-Chinese Translation of PainCQ Surveys

English Version of PainCQ Surveys	Chinese Version of PainCQ Surveys
PainCQ-I (Interdisciplinary Care)	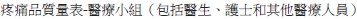
1.1 My healthcare team suggested approaches other than medications to help manage my pain. Examples are positioning my body, thinking about other things, deep breathing exercises, relaxation, and massage.	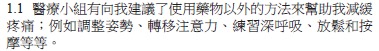
1.2 My healthcare team discussed my pain management plan with me.	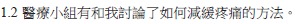
1.3 My healthcare team involved my family or significant other (friend) in the pain plan of care.	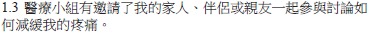
1.4 My healthcare team explained that patients will not become addicted to pain medication over time.	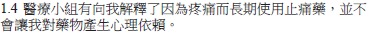
1.5 My healthcare team explained that taking pain medication may increase my activity level.	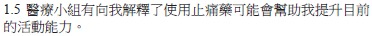
1.6 My healthcare team involved me in decisions about controlling my pain.	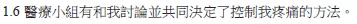
1.7 There was a team working together to make certain my pain was controlled.	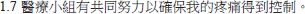
1.8 My doctors and nurses worked together to manage my pain.	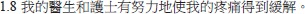
1.9 My healthcare team took time to discuss with me ways to manage my pain.	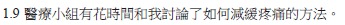
1.10 My healthcare team asked about how my pain affected my relationship with others.	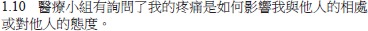
1.11 My healthcare team responded to changes in my pain.	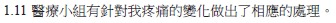
[New item] 1.12. My healthcare team explained that I should not stop or change the dose of the pain medication.	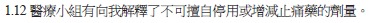
PainCQ-N (Nursing Care)	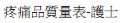
2.1 In addition to medications, my nurse suggested approaches to help manage my pain. Examples are positioning my body, thinking about other things, deep breathing exercises, relaxation, and massage.	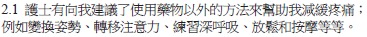
2.2 My nurse had a plan to treat my pain.	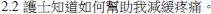
2.3 There was help available to manage my pain.	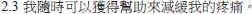
2.4 My nurse taught me that it is important to prevent the pain by taking the medication sooner rather than later.	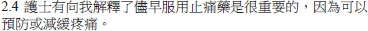
2.5 Approaches, in addition to medications, worked well to control my pain. Examples are positioning my body, thinking about other things, deep breathing exercises, relaxation, and massage.	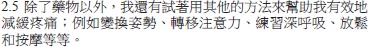
2.6 My nurse answered questions about my pain promptly.	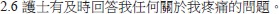
2.7 The pain medication kept me comfortable.	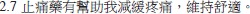
2.8 My nurse made sure I knew how to control my pain.	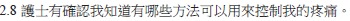
2.9 My nurse listened to me when I told him/her about my pain.	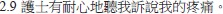
2.10 My nurse believed my reports about my pain.	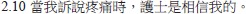
2.11 The pain medication worked quickly to ease my pain.	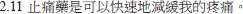
2.12 My nurse discussed side effects of the pain medications with me.	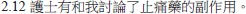
2.13 The pain medications worked well to control my pain.	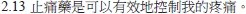
2.14 My nurse asked me about my pain.	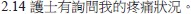
2.15 I had pain medication available when I needed it.	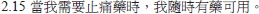
2.16 I felt comfortable talking to my nurse about my pain.	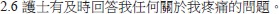
2.17 My nurse did a good job helping to control my pain.	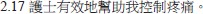
2.18 My nurse considered my pain when assisting me with movement and activity.	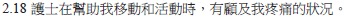
2.19 I felt confident that my pain could be controlled.	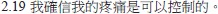
2.20 My pain was controlled.	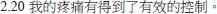
2.21 My nurse followed up to make certain that the pain medications were working.	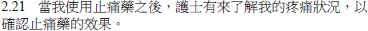
2.22 My requests for better pain relief were handled quickly.	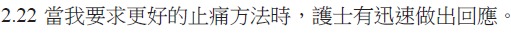

All 33 items on the PainCQ surveys were presented in the past tense, and “有” or “了” were used in the Chinese translation to indicate the past tense. Participants had no problem using the Likert-type scale in the C-PainCQ surveys, but recommended the addition of “not applicable or never happened” (“不適用或未曾發生”) as a response option. Overall, the participants agreed that most items from the prefinal version of C-PainCQ surveys were understandable and that some translations read awkwardly, especially for items in passive voice. For example, *my pain was controlled* was translated as “我的疼痛是有被控制” in the prefinal version of the C-PainCQ surveys; the translation perfectly reflected the English statement. Although this translation is understandable, it is not the way native Chinese speakers would say it. Therefore, this item was amended to “我的疼痛有得到了有效地控制” adding a few words to increase readability without changing the meaning (Table 2). Regarding the reading level of the final version of C-PainCQ surveys, the CRIE 3.0 indicated that this version has the reading level between the 4^th^ and 5^th^ grades of elementary school based on the education system in Taiwan, which may not be the same as other regions or countries.

## Discussion

This study translated the PainCQ surveys into Chinese following a cross-cultural adaptation approach. To evaluate whether the C-PainCQ could be used in different Chinese-speaking regions or countries, we included experts and participants from these areas (e.g., Taiwan, China, Hong Kong, and Singapore) when translating the PainCQ surveys and evaluating the C-PainCQ surveys. Because the prefinal version of the C-PainCQ surveys was produced by the translators with high levels of education, it was important to have potential users with less education to review it to improve the readability of the C-PainCQ surveys.

About half of the participants in Group 1 indicated their first language as Taiwanese. Taiwanese is a spoken language with no corresponding written language. This was not a concern as they were fluent in reading and speaking Chinese. For Group 2 participants who evaluated semantic equivalence between the prefinal version of C-PainCQ surveys and the English version of PainCQ surveys, five participants were born in Taiwan but none of them indicated their first language was Taiwanese.

Consistent with previous studies, we found that English statements with passive voice were challenging to translate (Guo, 2016; Tsai et al., 2018). Although the participants could interpret the translation as expected by way of keywords in the statement, they suggested to add words which are not in the source language to improve readability for the translation. However, the researcher needs to carefully evaluate whether the additional words distort the meaning of the source language.

*My nurse* in Chinese translation could be interpreted as primary nursing care model to some of our participants. By dropping *my* from the Chinese translation, the meaning of *nurse* in the C-PainCQ surveys broadly implies nurses in different nursing care models, such as primary, functional, or team nursing care models. This translation reflects the patients’ experience and remains the meaning of evaluating quality of pain care provided by nurses.

One new item related to education about pain medication compliance was added in the C-PainCQ Interdisciplinary Care survey. This item reflected concerns about use of medications and fear of addictions which was reported as one barrier for effective pain management in Chinese population (Xu et al., 2018). How this item fits into the C-PainCQ surveys warrants further examination. For example, in future research, we can use factor analysis to investigate the loading of this new item in the PainCQ Interdisciplinary Care survey. The item related to addiction (Item 1.4) has also been challenging in the United States due to the opioid epidemic and has been deleted in recent use of the English Pain-CQ surveys (Rice et al., 2019). The item related to medication compliance may more appropriately address the requisite care related to medication use.

Chinese speakers from different regions or countries have different education systems. Because the CRIE was developed in Taiwan, the result of the reading level for the C-PainCQ surveys might only generalize to people receiving education in Taiwan.

## Conclusion

Translating the PainCQ into Chinese is needed to gain understanding of the perceived quality of pain care among non-English-speaking Chinese-Americans or other Chinese speakers. Instead of generating several Chinese versions of PainCQ surveys, this study aims to generate one Chinese version of the PainCQ surveys which can be used in different Chinese speaking regions or countries by switching between traditional and simplified Chinese characters. The C-PainCQ surveys generated in this study provide a good foundation for the next step of evaluating reliability and construct validity with a large sample.

## Acknowledgments

The authors would like to express their gratitude to our team of research assistants: Szu-Huei Su, Rumei Yang, and Yin-Shun Chiu.

## Declaration of Conflicting Interests

The authors declared no potential conflicts of interest with respect to the research, authorship, and/or publication of this article.

## Funding

This study is funded by Oncology Nursing Foundation Research Career Development Award, USA, University Research Committee (URC) from the University of Utah, USA, and Chi Mei Medical Center, Taiwan (# CMFHR10364).
